# Supplementary material for: Modeling glioblastoma heterogeneity as a dynamic network of cell states
Source: Mol Syst Biol. 2021 Sep 16;17(9):e10105. doi: 10.15252/msb.202010105 (PMC8444284; doi:10.15252/msb.202010105)
Supplement: Supplementary file 6 — Source Data for Figure 5 [file MSB-17-e10105-s004.zip › Figure5A_sourcedata/GSEA_3017/hallmarks_stateC.GseaPreranked.1621934520804/HALLMARK_CHOLESTEROL_HOMEOSTASIS.html]

Details for gene set HALLMARK\_CHOLESTEROL\_HOMEOSTASIS[GSEA]

|  || Dataset | state13017 |
| Phenotype | NoPhenotypeAvailable |
| Upregulated in class | na\_neg |
| GeneSet | HALLMARK\_CHOLESTEROL\_HOMEOSTASIS |
| Enrichment Score (ES) | -0.54658103 |
| Normalized Enrichment Score (NES) | -2.2814248 |
| Nominal p-value | 0.0015948963 |
| FDR q-value | 0.0025795787 |
| FWER p-Value | 0.008 |
Table: GSEA Results Summary

  

Fig 1: Enrichment plot: HALLMARK\_CHOLESTEROL\_HOMEOSTASIS      
 Profile of the Running ES Score & Positions of GeneSet Members on the Rank Ordered List

  

| PROBE | GENE SYMBOL | GENE\_TITLE | RANK IN GENE LIST | RANK METRIC SCORE | RUNNING ES | CORE ENRICHMENT || 1 | FDFT1 |  |  | 243 | -0.262 | -0.3887 | No |
| 2 | SQLE |  |  | 293 | -0.274 | -0.4410 | No |
| 3 | DHCR7 |  |  | 322 | -0.285 | -0.4556 | No |
| 4 | MVD |  |  | 333 | -0.288 | -0.4388 | No |
| 5 | S100A11 |  |  | 361 | -0.301 | -0.4499 | No |
| 6 | HMGCR |  |  | 418 | -0.334 | -0.5071 | Yes |
| 7 | LDLR |  |  | 419 | -0.334 | -0.4677 | Yes |
| 8 | IDI1 |  |  | 451 | -0.353 | -0.4796 | Yes |
| 9 | ALCAM |  |  | 467 | -0.370 | -0.4617 | Yes |
| 10 | LGALS3 |  |  | 471 | -0.374 | -0.4227 | Yes |
| 11 | CYP51A1 |  |  | 476 | -0.378 | -0.3849 | Yes |
| 12 | SCD |  |  | 504 | -0.431 | -0.3807 | Yes |
| 13 | CD9 |  |  | 515 | -0.443 | -0.3456 | Yes |
| 14 | HMGCS1 |  |  | 529 | -0.466 | -0.3130 | Yes |
| 15 | TNFRSF12A |  |  | 550 | -0.519 | -0.2862 | Yes |
| 16 | CLU |  |  | 581 | -0.690 | -0.2566 | Yes |
| 17 | LPL |  |  | 593 | -0.885 | -0.1710 | Yes |
| 18 | FABP5 |  |  | 596 | -1.477 | -0.0000 | Yes |
Table: GSEA details [plain text format]

  

Fig 2: HALLMARK\_CHOLESTEROL\_HOMEOSTASIS: Random ES distribution      
 Gene set null distribution of ES for **HALLMARK\_CHOLESTEROL\_HOMEOSTASIS**
